# Establishment of an Adult Medaka Fatty Liver Model by Administration of a Gubra-Amylin-Nonalcoholic Steatohepatitis Diet Containing High Levels of Palmitic Acid and Fructose

**DOI:** 10.3390/ijms22189931

**Published:** 2021-09-14

**Authors:** Koichi Fujisawa, Taro Takami, Shoki Okubo, Yuto Nishimura, Yusaku Yamada, Keisuke Kondo, Toshihiko Matsumoto, Naoki Yamamoto, Isao Sakaida

**Affiliations:** 1 Department of Liver Regenerative Medicine, School of Medicine, Yamaguchi University, Minami Kogushi 1-1-1, Ube Yamaguchi 755-8505, Japan; fujisawa@yamaguchi-u.ac.jp; 2 Department of Gastroenterology and Hepatology, Graduate School of Medicine, Yamaguchi University, Minami Kogushi 1-1-1, Ube Yamaguchi 755-8505, Japan; i097eb@yamaguchi-u.ac.jp (Y.Y.); g041eb@yamaguchi-u.ac.jp (K.K.); tm0831@yamaguchi-u.ac.jp (T.M.); nao-yama@yamaguchi-u.ac.jp (N.Y.); sakaida@yamaguchi-u.ac.jp (I.S.); 3 Department of Laboratory Science, School of Medicine, Yamaguchi University, Minami Kogushi 1-1-1, Ube Yamaguchi 755-8505, Japan; g004ed@yamaguchi-u.ac.jp (S.O.); g022ed@yamaguchi-u.ac.jp (Y.N.); 4 Department of Oncology and Laboratory Medicine, Graduate School of Medicine, Yamaguchi University, Minami Kogushi 1-1-1, Ube Yamaguchi 755-8505, Japan; 5 Health Administration Center, Yamaguchi University, Minami Kogushi 1-1-1, Ube Yamaguchi 755-8505, Japan

**Keywords:** hepatic steatosis, medaka, fructose, ballooning

## Abstract

Among lifestyle-related diseases, fatty liver is the most common liver disease. To date, mammalian models have been used to develop methods for inhibiting fatty liver progression; however, new, more efficient models are expected. This study investigated the creation of a new model to produce fatty liver more efficiently than the high-fat diet medaka model that has been used to date. We compared the GAN (Gubra-Amylin nonalcoholic steatohepatitis) diet, which has been used in recent years to induce fatty liver in mice, and the high-fat diet (HFD). Following administration of the diets for three months, enlarged livers and pronounced fat accumulation was noted. The GAN group had large fat vacuoles and lesions, including ballooning, compared to the HFD group. The GAN group had a higher incidence of lesions. When fenofibrate was administered to the fatty liver model created via GAN administration and liver steatosis was assessed, a reduction in liver fat deposition was observed, and this model was shown to be useful in drug evaluations involving fatty liver. The medaka fatty liver model administered with GAN will be useful in future fatty liver research.

## 1. Introduction

Lifestyle factors, such as dietary habits, exercise, smoking, and alcohol consumption, greatly contribute to the onset and progression of lifestyle-related diseases, such as diabetes, obesity, hyperlipidemia, and hypertension. Among the lifestyle-related diseases, fatty liver caused by over nutrition is the most common liver disease, with the number of affected patients increasing continuously. Fatty liver disease is the collective name for liver damage caused by fat deposits in hepatocytes. When no clear history of alcohol use exists, it is referred to as nonalcoholic fatty liver disease (NAFLD). NAFLD is classified into simple fatty liver, which has a good prognosis, and nonalcoholic steatohepatitis, which has been reported to progress over time to cirrhosis, and liver cancer [1]. Thus far, to develop methods of inhibiting fatty liver progression, mice have been used; however, new, more efficient models are required [2].

In fatty liver animal models, it is known that a high-fat diet (HFD) and AMLN (amylin liver non-alcoholic steatohepatitis) diet have been administered to mice. Given that trans-fatty acids used in the AMLN diet have recently been prohibited by the FDA (US Food Drug Administration. 2018. Final determination regarding partially hydrogenated oils (removing trans-fat). Available online: https://www.federalregister.gov/documents/2018/05/21/2018-10714/final-determination-regarding-partially hydrogenated-oils (accessed on 28 January 2021)), the Gubra-Amylin-nonalcoholic steatohepatitis (GAN) diet, which uses non-trans fats and contains palmitic acid and fructose additives with nutritional elements similar to the AMLN diet, was created. It has been reported to exhibit the same effects as the AMLN diet [3].

Medaka are small in size and easy to feed, making it possible to perform experiments using a large number of individuals at a lower cost than using mice or rats [4]. In addition, medaka have been studied for a long time, their inbred strains have been established, and they are suitable for the analysis of liver fat metabolism because they tend to build up fat on their liver for overwintering [5,6]. We previously reported the utility of the medaka model fed a high-fat diet for tissue and lipid analysis [7]. Furthermore, as a noninvasive method of analyzing fatty liver progression due to HFD liver steatosis has been evaluated using direct observation of transparent medaka or using ultrasound [8]. Changes in liver steatosis due to alcohol administration have also been evaluated using direct observation and ultrasound in transparent medaka [9]. Fatty liver studies with medaka have all used the HFD diet; however, there have been no reports on the effectiveness of the GAN diet. Here, we aimed to create a more efficient medaka fatty liver model and to perform a comparative study on high-fat and GAN diets.

## 2. Results

### 2.1. Evaluation of Body Weight, Liver Weight, and Liver–Weight Ratio in Each Dietary Group

**Figure 1:**
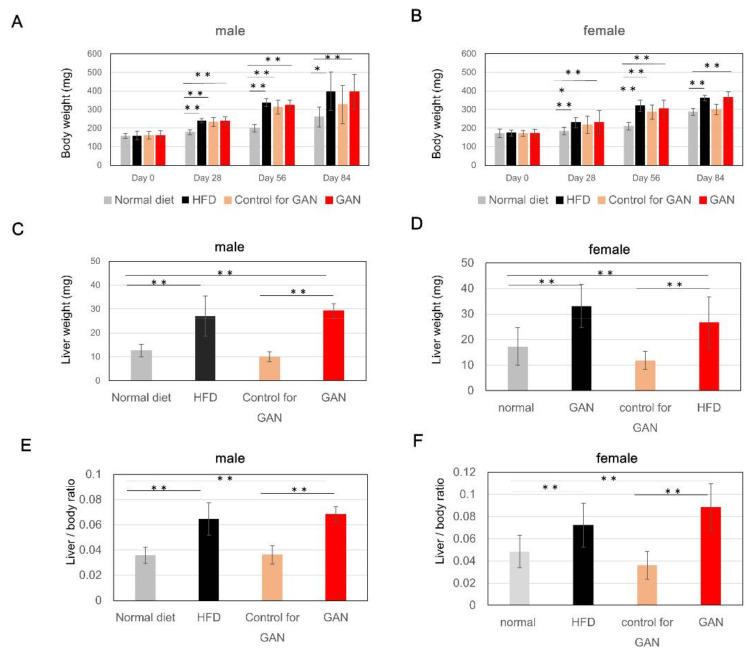
Body weight, weight of liver, and liver–weight ratio after administration of each diet. (**A**,**B**) Changes in body weight after administration of each feed; (**C**,**D**) Weight of liver three months after administration of each diet; (**E**,**F**) Liver–weight ratio three months after administration of each diet; Data are represented as the mean ± standard deviation (SD) (*n* = 10); * indicates significant difference (*p* < 0.05), ** indicates significant difference (*p* < 0.01).

**Figure 2:**
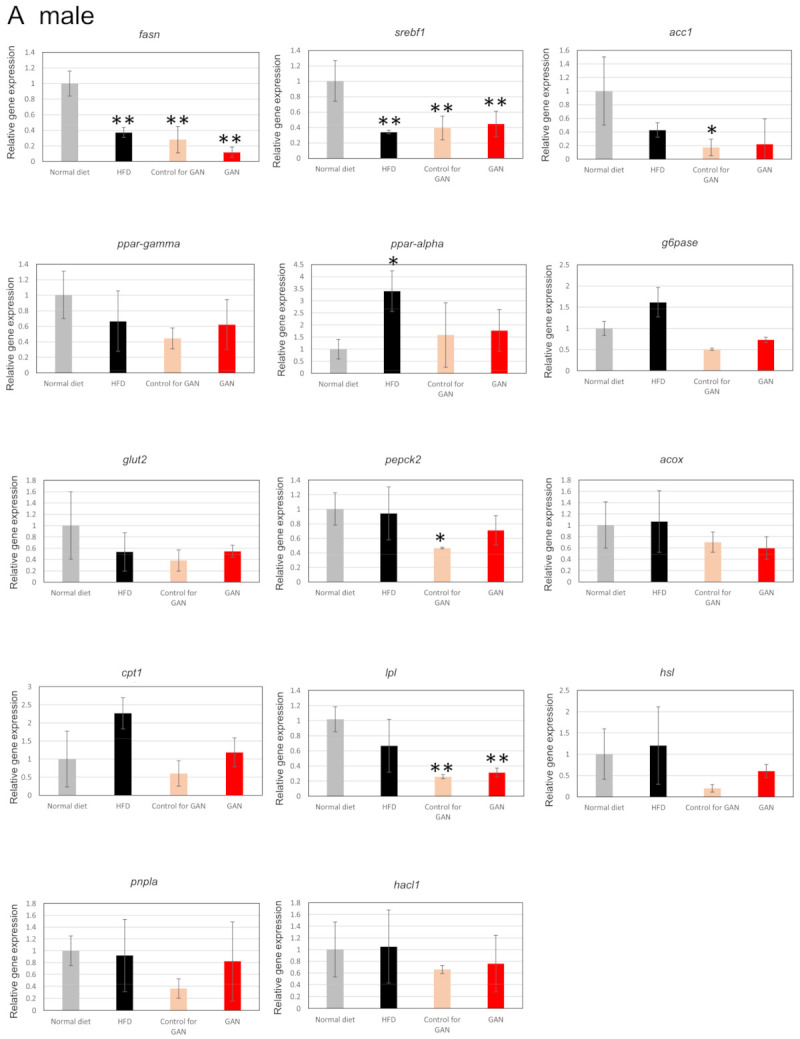

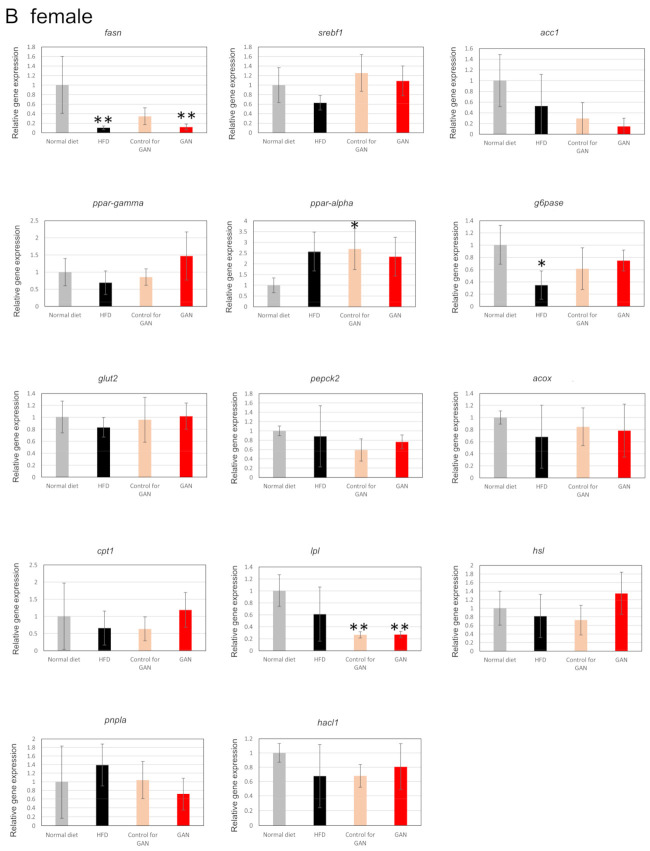
Expressional analysis of medaka genes by real-time RT PCR. (**A**): male; (**B**): female. Data are represented as the mean ± standard deviation (SD), (*n* = 4); * indicates significant difference (*p* < 0.05), ** indicates significant difference (*p* < 0.01). *fasn*: fatty acid synthase, *srebf1*: sterol regulatory element binding transcription factor 1, *acc1*: phospho-acetyl CoA Carboxylase 1, *ppar-gamma*: Peroxisome Proliferator-Activated Receptor γ, *ppar-alpha*: peroxisome proliferator-activated receptor α, *g6pase*: glucose-6-phosphatase, *glut2*: glucose transporter 2, *pepck2*: phosphoenolpyruvate carboxykinase2, *acox*: acyl-CoA oxidase, *cpt1*: carnitine palmitoyltransferase 1, *lpl*: lipoprotein lipase, *hsl*: hormone sensitive lipase, *pnpla2*: phospholipase domain-containing protein 2, *hacl1*: 2-hydroxyacyl-CoA lyase 1.

Changes in body weight were measured at 1, 2, and 3 months after the start of the experiment. Compared to the normal diet group, both males and females in the HFD group, the control for GAN group, and the GAN group exhibited a tendency to gain weight (Figure 1A,B). The control group had a smaller weight increase than the HFD and GAN groups. The liver weight (Figure 1C,D), and liver–weight ratio (Figure 1E,F) of the control group were similar those of the normal diet group; however, increases were observed in the HFD and GAN groups. The gene expression level of the fat metabolism related genes by real-time RT PCR was evaluated in the control group, the HFD group, the control for GAN group, and the GAN group (Figure 2).

### 2.2. Macroscopic Findings in the Liver

**Figure 3:**
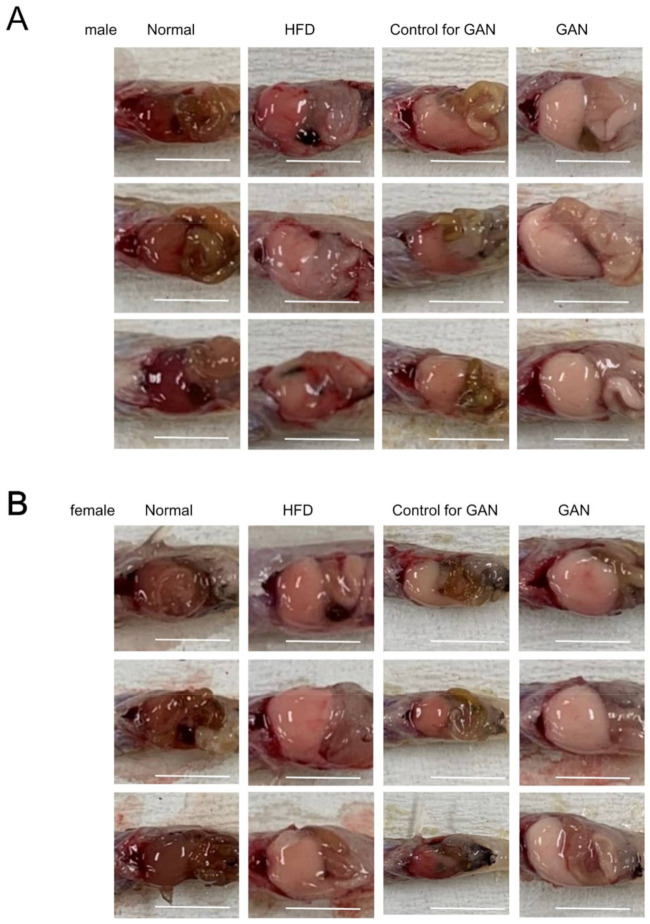
Liver changes in medaka three months after the start of the experiment. (**A**) Representative liver macroscopic findings (male) after administering each diet for three months. (**B**) Representative liver macroscopic findings (female) after administering each diet for three months. The scale bar represents 5 mm.

Livers of both male and female mice in the HFD and GAN groups developed a white tone and were greatly enlarged compared to those in the remaining control groups. Compared to the normal group, livers of the control group had a white tone; however, no changes in size were observed (Figure 3A,B).

### 2.3. Histological Evaluation of the Liver

**Figure 4:**
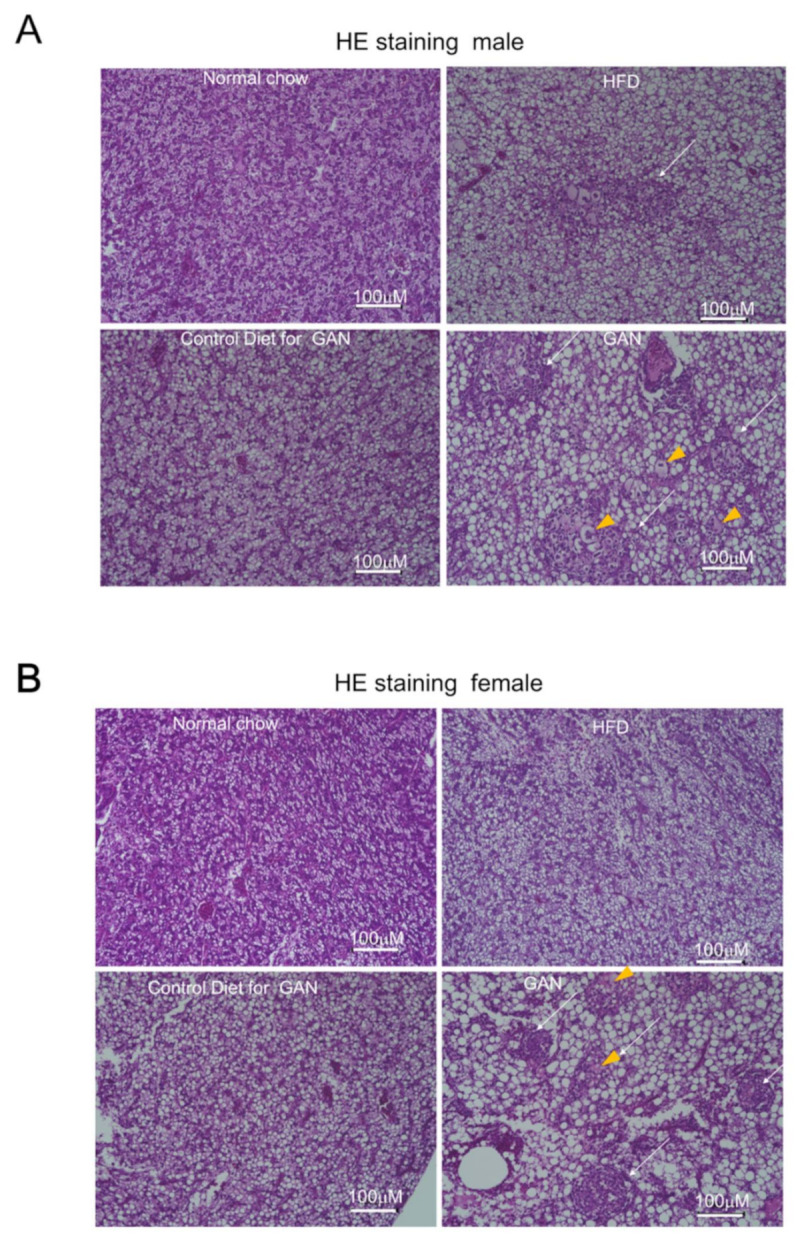
Evaluation of microscopic changes in the liver using hematoxylin and eosin (HE) staining. (**A**) male, (**B**) female. The arrowhead indicates ballooning cells; the arrow represents inflammatory infiltration; the white scale bar represents 100 µm.

**Figure 5:**
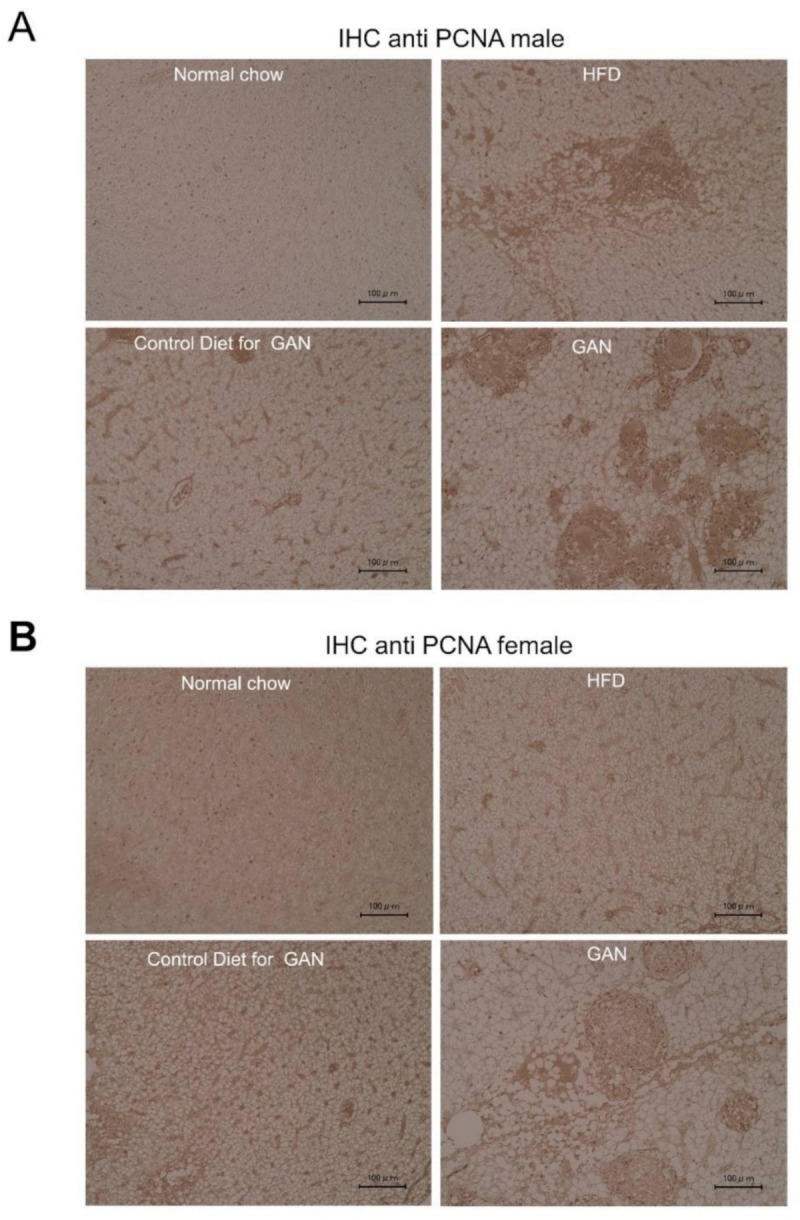
Evaluation of PCNA expression in the liver by immunohistochemistry. (**A**) male, (**B**) female. Anti-PCNA antibodies. The black scale bar represents 100 µm.

Evaluation of livers using hematoxylin and eosin (HE) staining revealed many more fat vacuoles in the control group than the normal group, and livers of the HFD and GAN groups were found to have discernibly larger and more numerous fat vacuoles. Furthermore, a greater number of large fat vacuoles was found in the GAN group than the HFD group, around ballooning cell lesions, in which inflammatory cell aggregates could be seen. In males, these lesions were found in seven of the nine specimens in the GAN group, five of the nine specimens in the HFD group, and zero of the nine specimens in the normal and control groups. In females, they were found in eight of the nine specimens in the GAN group, three of the nine specimens in the HFD group, and zero of the nine specimens in the normal and control groups (Figure 4A,B, Table 1). When PCNA antibodies were used for immunohistochemistry, many cells were found with PCNA present in the nucleus (Figure 5A,B).

### 2.4. Evaluation of the Effects of Fatty Liver Improvement by the Pparα Activator, Fenofibrate

**Figure 6:**
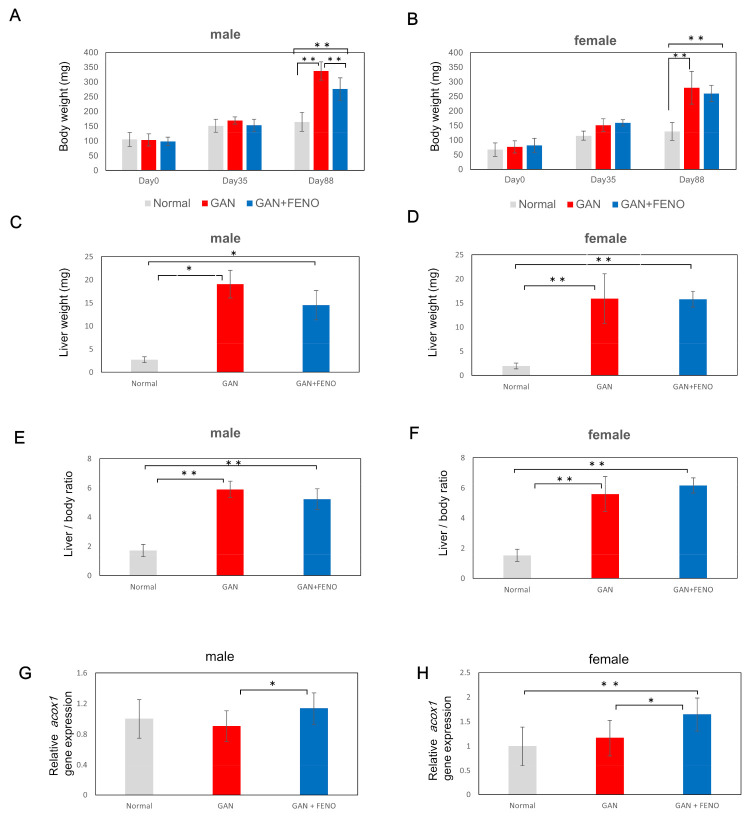
Changes in body weight, liver weight, and liver–weight ratio due to fenofibrate administration. (**A**,**B**) Changes in body weight after administration of each feed; (**C**,**D**) Liver weight three months after administration of each diet; (**E**,**F**) Liver–weight ratio three months after administration of each diet; (**G**,**H**) Gene expressional analysis of *acox1*; (**A**,**C**,**E**,**G**): male; (**B**,**D**,**F**,**H**): female. Data are represented as the mean ± standard deviation (SD) (*n* = 10). * indicates significant difference (*p* < 0.05), ** indicates significant difference (*p* < 0.01).

To verify whether the fatty liver model produced using GAN administration is suitable for drug evaluation, fenofibrate, whose effectiveness against fatty liver has been established to date in mammals, was used. In males, a significant reduction in body weight was found in the fenofibrate group compared to that in the GAN-treated group. In addition, there was no significant difference (*p* < 0.05) in liver weight or liver–weight ratio; however, a decreasing trend was observed (Figure 6A,C,E). In females, no significant reduction in body weight, liver weight, or liver–weight ratio was found in the fenofibrate group compared to the GAN group; however, a decreasing trend was observed (Figure 6B,D,F). We evaluated the gene expression of acyl-CoA oxidase-1 (*acox1*), a Pparα target gene. Changes in *acox1* were induced by fenofibrate administration (Figure 6G,H).

**Figure 7:**
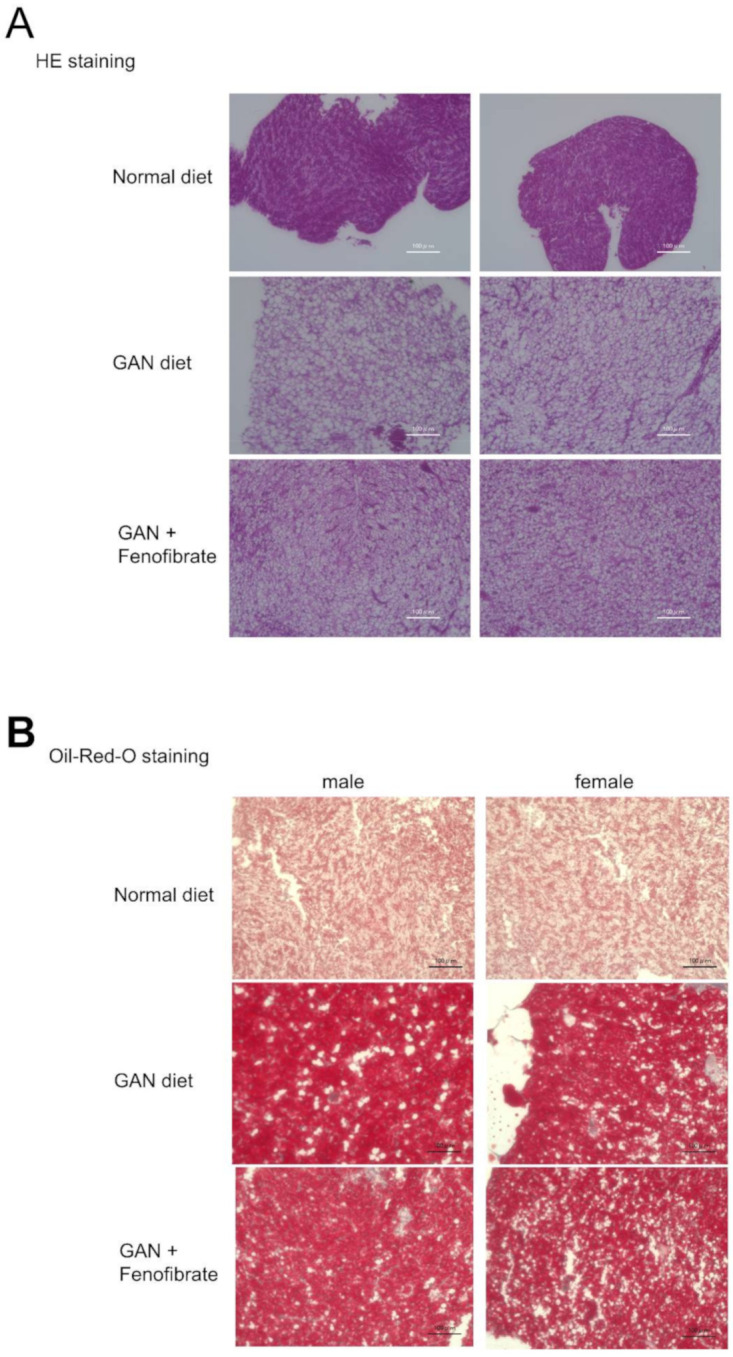
Liver evaluation using each type of staining after three months of fenofibrate administration. (**A**) Evaluation using hematoxylin and eosin (HE) staining, (**B**) Evaluation with Oil Red O staining. The scale bar represents 100 µm.

Livers of both male and female mice in the GAN and fenofibrate groups were greatly enlarged compared to the normal group. In both males and females, HE staining revealed a reduction in the size of fat vacuoles in the fenofibrate group compared to the non-fenofibrate group. However, in both males and females, lesions in which inflammatory cell aggregates could be seen were present around ballooning cells; however, no differences in lesions and their sizes or numbers were found (Figure 7A, B).

## 3. Discussion

This study did not determine clear differences in body weight and liver weight between the HFD group and the GAN group; however, clear differences in liver steatosis were observed. To date, it has been reported that when mice are administered a GAN diet, obesity, steatosis, lobular inflammation, and hepatocellular ballooning occur, and it is effective in creating a fatty liver model [3]. It has been proposed that GAN contains palm oil and fructose, and the fatty acid composition of palm oil is composed of 50% saturated fatty acids (palmitic acid 43%, stearic acid 4%). For a long time, the question of whether the accumulation of fat in liver cells exerts a protective effect or an injurious effect on liver cells has existed. At present, it is thought that the accumulation of neutral fat is in itself protective; however, fatty acids, including saturated ones and exposure to their metabolites, are injurious to liver cells [10]. It has been reported that saturated free fatty acids, particularly palmitic acid and stearic acid, induce apoptosis and inflammation through a variety of mechanisms and have strong lipotoxicity, which activates signal pathways and death receptors, and causes an adverse effect on cells owing to multiple mechanisms such as endoplasmic reticulum (ER) stress, mitochondrial function irregularities, and oxidative stress [11].

Overconsumption of fructose increases the risk of nonalcoholic fatty liver disease; however, its mechanism is unclear. Until recently, fructose was thought to be absorbed in the intestines and immediately transported to the liver and metabolized. However, it has been reported that fructose metabolism occurs almost entirely in the small intestine and is first phosphorous-oxidized by the ketohexokinase present in the small intestine, which then proceeds through the downstream pathway, and the small intestine acts to prevent fructose from being transported directly to the liver [12]. The intestinal barrier is made up of tightly packed epithelial cells that prevent the outflow of bacteria and bacterial products such as endotoxins. If there is excessive fructose, this barrier becomes damaged owing to a reduction in the protein production that maintains it [13]. It has been reported that *Lachnospira*, *Parasutterella*, *Marvinbryantia*, and *Blantia* in the large intestine are increased by fructose administration [14]. Moreover, it is believed that the formation of butyrate and glycerate, which are hepatotoxic metabolites, in the small intestine or by intestinal microbiota is a factor in fatty liver deterioration; however, further study is required [12].

When the fatty liver model created by GAN administration was evaluated for suitability for drug evaluation, a reduction in liver fat deposition owing to fenofibrate administration was demonstrated; hence, fenofibrate utility was demonstrated in this model. Fenofibrate induces lipoprotein lipase expression at the transfer level by activating *Ppar*α, thereby stimulating the metabolism of the lipoprotein rich in triglycerides (TG) and promoting fatty acid oxidation of the hepatocyte mitochondria. This synthesizes TG and very low-density lipoprotein and produces secretions [15]. Another report showed that fenofibrate increases levels of high-density lipoprotein by inducing the expression of Apo A1 and A2 [16], and its effectiveness against fatty liver has been demonstrated [17]. It is speculated that the body weight and liver weight reduction tendencies in the present study are caused by the accentuation of the fatty acid oxidation differentiation process due to the *Ppar*α activation action of fenofibrate.

Regarding inflammatory cells inside the liver and lesions that contain ballooned cells, nonalcoholic steatohepatitis is characterized by inflammatory infiltration and hepatocyte ballooning, and it has recently been reported that characteristic microenvironments (hepatic crownlike structures) are formed in the liver and tissue-resident macrophages (Kupffer cells) play an important role, leading to the onset of nonalcoholic steatohepatitis [18]. In this study, inflammatory infiltration was observed in the medaka model, and it is believed that it migrated in response to hepatocyte disturbance. It is said that TNFα or CD11β and F4/80 positive cells are involved in mammals [19]; however, in medaka, there are few usable antibodies, and it is difficult to identify the types of inflammatory cell infiltration. It is anticipated that future research will address this question. Moreover, in comparison to mammals, liver fibrosis was almost entirely absent; hence, it is necessary to be mindful of the differences in the pattern of progression of liver damage. However, we believe that medaka are highly useful for lipid metabolism research owing to their low cost, ability to handle a large number of individuals, and because genetically modified individuals can be easily obtained using CRISPR/CAS9. Thus, we anticipate more fruitful research in future.

This study succeeded in producing a highly efficient medaka liver lipid metabolism model utilizing the GAN diet, which has not previously been used with medaka. We also confirmed the drug efficacy of fenofibrate, which is known as an effective drug. We believe that this model will be useful in future fatty liver research, such as drug screening.

## 4. Materials and Methods

### 4.1. Animals

This study used a 6–10 month-old inbred medaka strain (Kyoto-Cab) [20]. All fish were cared for in compliance with the Animal Care Guidelines of Yamaguchi University (Yamaguchi City, Yamaguchi Prefecture, Japan) (Approval No. 21-038). During the experiment, fish were raised in plastic aquariums covered with plastic covers and provided fluorescent light from 08:00 to 20:00. The water in the aquarium was maintained at 26 °C. The light source was fluorescent (20 W, National, Tokyo, Japan), and the strength of the light on the water surface was adjusted to 1500–2000 lux. Medaka were sacrificed on ice. Following the dissection of their abdomens in the middle, their livers were necropsied.

### 4.2. Diets

Four types of feed were used: normal chow(Hikari Crest; Kyorin Co. Ltd., Hyogo, Japan), a pet feed administered to ordinary medaka; HFD (HFD32; CLEA Japan Inc., Tokyo, Japan), a HFD administered to mice; GAN (D09100310, EP Trading, Tokyo, Japan), which contains a large amount of palm oil and fructose and has recently been used to induce nonalcoholic steatohepatitis in mice; and a control for the GAN diet (D09100304, EP Trading). The proportions of protein, sugars, and fats in each feed are shown in Table 2.

### 4.3. Dietary Administration Tests

A total of 80 Cab medaka (40 male and 40 female) were prepared. The medaka were selected such that there were 10 males and 10 females of the same weight, and a total of 20 medaka were divided into four groups and placed into four aquariums. The groups were the normal diet group, the HFD diet group, the control for the GAN group, and the GAN diet group. The body weights of medaka in all four groups were measured monthly. Following three months of feeding, body weight and necropsied liver weight were measured, and the condition of the livers was observed macroscopically and histologically, and differences according to each feed were confirmed.

### 4.4. Immunostaining

Immunostaining was performed according to a previously described method [21]. A simplified description of this method is presented here. Liver tissues were stabilized in Bouin’s fluid, embedded in paraffin, and cut into 3 μm slices. Deparaffinization was performed using Remozole and ethanol, and endogenous peroxidase was blocked for 30 min at room temperature in fresh 0.3% hydrogen peroxide in methanol. Thereafter, antigen activation was carried out using microwaves at 95 °C in 10 mM sodium citrate buffer solution for 6 min. Normal goat serum (Vector Laboratories; Burlingame, CA, USA) was applied for 20 min and subsequently removed. The slices were diluted 1:300 with mouse anti-PCNA antibodies (clone PC10, Sigma, St. Louis, MO, USA) and incubated overnight at 4 °C in a damp chamber. The slices were then washed three times in phosphate-buffered saline (PBS), and incubated with biotinylated secondary antibodies for 1 h at room temperature. Binding antibodies were detected using the avidin-biotin complex immunoperoxidase technique (Vector Laboratories, Burlingame, CA, USA).

### 4.5. Real-Time RT-PCR

The total RNA was isolated from six fish each time using Isogen (Life Technology, Tokyo, Japan) according to the manufacturer’s instructions. For cDNA synthesis, Taqman reverse transcription reagents (Roche Diagnostics, Indianapolis, IN, USA) were used as described in the manufacturer’s manual. Variations in the expression of the genes, and the control *Ef1α* gene, were analyzed using a Step One Plus real-time PCR system (Life Technologies, Tokyo, Japan) with SYBR green. For the RT-PCR analysis, primers were chosen for their dissociation curves, lack of non-specific amplification, and relatively good amplification efficiency. The base sequences for the utilized primers are as follows: *fasn* Forward 5′-TGGAAGCTCTGGGTCACTCT-3′, Reverse 5′-GACAGGGACAGTTCCAATAC-3′; *srebf1* Forward 5′- GCCCTCCTGAACGATATTGA -3′, Reverse 5′- AAACGTCGGTAGCTTCTCCA -3′, *acc1* Forward 5′-GAGTGACGTCCTGCTTGACA-3′, Reverse 5′- ACCTTTGGTCCACCTCACAG-3′, *PPARgamma* Forward 5′-AAGACCACGGAGATCAAGTTCAGG -3′, Reverse 5′-ATCTCTCGCTCCAGAGTTGAGGTCT-3′, *PPARalpha* Forward 5′-GCTTTGTTCGTAGCCACCAT-3′, Reverse 5′-GGACCTTCACGATGTTCTCC-3′, *g6pase* Forward 5′-GGTTTGCATGTCCAGGGTCT-3′, Reverse 5′- GGGCTTTGTCCAGAGTCCAA-3′, *glut2* Forward 5′- GCCCCCGGTACCTTTACATC-3′, Reverse 5′-CGTATCACACGGCCCCTTTA -3′, *pepck2* Forward 5′-GTCGGCCTTTACCTCTCACA-3′, Reverse 5′-ACAGGATCTGCCTGGTGTCT -3′, *acox1* Forward 5′-ACAAGAGCATGGTCACAGGC-3′, Reverse 5′-GGCAGCCATTTGCTCATCTG-3′, *cpt1* Forward 5′-ATGTCTACCTCCGTGGACGA -3′, Reverse 5′-CAAGTTTGGCCTCTCCTTTG-3′, *lpl* Forward 5′-GCTCGGAGCACCAAGATGTA-3′, Reverse 5′-CTCCCCGTGAGTTCCAAACA-3′, *hsl* Forward 5′-CCTCCTGAGCAGAGGAGACA-3′, Reverse 5′-GACTCGACCAATCGCTTTGC-3′, *pnpla* Forward 5′-ACGCTTCCAGATGATTGCCA-3′, Reverse 5′-CCTTGAAGCGTGGGAGGAAT-3′, *hacl1* Forward 5′-ATGCTCGCTACGATCAGGTG-3′, Reverse 5′-GAGCCATGGAAAGTCCTGCT-3′, *ef1α* Forward 5′-AACACTCCTTGAAGCTCTTGTACC-3′, Reverse 5′-AATCGCTCCACCAACTAAGAACGGCCATGC-3′.

### 4.6. Statistical Methods

Normality tests were carried out on all groups (body weight, liver weight, and liver–weight ratio) with a significance level of 0.05, and dispersion tests were performed using Bartlett’s test. If all groups showed normal distribution and equal variances, testing was performed using the Tukey–Kramer parametric method. In all other cases, testing was performed using the Steel–Dwass nonparametric method.

## Figures and Tables

**Table 1:** Frequency and number of lesions.

Diet	Gender	Number of Specimens with Lesions/Number of Specimens
Normal	male	0/9
	female	0/9
HFD	male	5/9
	female	3/9
Control for GAN	male	0/9
	female	0/9
GAN	male	7/9
	female	8/9

**Table 2:** Composition of diets used in the experiment.

Diet	Normal Chow	HFD	Control for GAN	GAN
Protein (cal%)	62	20	20	20
Carbohydrate (cal%)	13	23	70	40
Fat (cal%)	25	57	10	40
kcal/100g	325	508	380	450
Fructose (%/g)	0	0	0	22
Parm oil (cal%)	0	0	0	15

HFD, high-fat diet; GAN, Gubra-Amylin nonalcoholic steatohepatitis.

## Data Availability

Not applicable.

## Abbreviations

α-SMA	α-smooth muscle actin
GAN	Gubra-Amylin-nonalcoholic steatohepatitis
HFD	High fat diet
IHC	Immunohistochemistry
HSC	Hepatic stellate cell
IPA	Ingenuity Pathway Analysis
PCNA	Proliferating Cell Nuclear Antigen
SAGE	Serial analysis of gene expression
